# Twenty-five years of surveillance for familial and hereditary pancreatic ductal adenocarcinoma: Historical perspectives and introduction to the special issue

**DOI:** 10.1007/s10689-024-00404-0

**Published:** 2024-06-06

**Authors:** Hans FA Vasen, Marcia Irene Canto, Michael Goggins

**Affiliations:** 1 Department of Gastroenterology & Hepatology, Leiden University Medical Center, Leiden, The Netherlands; 2 Department of Medicine, Division of Gastroenterology, The Sol Goldman Pancreatic Cancer Research Center, Johns Hopkins Medical Institutions, Baltimore, MD USA; 3 Department of Pathology, and Oncology, The Sol Goldman Pancreatic Cancer Research Center, Johns Hopkins Medical Institutions, Baltimore, MD USA

**Keywords:** Pancreatic ductal adenocarcinoma, Genetics, Familial pancreatic cancer, History, Surveillance, Early detection

## Abstract

In the 1990s, as prevention became a central strategy in the battle against cancer and the molecular genetics revolution uncovered the genetic basis of numerous hereditary cancer syndromes, there were no options available for patients at increased risk of developing pancreatic cancer. When surveillance efforts for those at familial and hereditary risk of pancreatic cancer emerged in the late 1990s, it was uncertain if early detection was achievable.

In this introduction to the special issue, we offer an overview of the history of surveillance for pancreatic cancer, including the first reports of familial pancreatic cancer in the medical literature, the initial results of surveillance in the United States and the initiation of surveillance programs for hereditary pancreatic cancer in the Netherlands.

This special issue features a collection of 18 articles written by prominent experts in the field, focusing specifically on refining surveillance methodologies with the primary objective of improving care of high-risk individuals. Several reviews in this collection highlight improved survival rates associated with pancreas surveillance, underlying the potential of early detection and improved management in the continuing fight against pancreatic cancer.

## Cancer prevention and molecular genetics revolution in the 1990s

The ongoing battle against cancer has been a global health endeavor for decades. In 1997, Bailar et al. published a comprehensive analysis of efforts to combat cancer between 1970 and 1994 [1]. Despite the introduction of novel treatments during this period, there were minimal reductions in cancer-related mortality rates. The authors advocated for a paradigm shift towards prevention as the most promising approach to cancer control. Subsequently, in addition to tobacco control efforts, the 1990s saw the launch of population-based screening initiatives targeting prevalent cancers such as breast and colorectal cancer. Screening for pancreatic cancer was deemed inappropriate due to its low prevalence and the absence of accurate non-invasive screening tools.

In parallel, the field of cancer research experienced a revolution due to major advances in molecular genetics. The genetic basis of various hereditary cancer syndromes was unraveled during this period, including hereditary breast cancer (*BRCA1, BRCA2)*, Lynch syndrome (mismatch repair genes), Familial Adenomatous Polyposis (FAP) *(APC*), Familial Atypical Multiple Mole Melanoma (FAMMM)(*CDKN2A*), and Peutz-Jeghers syndrome (*STK11*). Many of these genetic aberrations were found to be associated with pancreatic cancer [2]. As delineated by Jacobs et al. in this issue [3], the highest risks of developing pancreatic cancer were observed in individuals with a pathogenic germline variant in *CDKN2A* (15–20%), *STK11* (11–36%) and *PRSS1* (10–70%), while lower risks (less than 10%) were reported for carriers of a pathogenic germline variant in *BRCA1, BRCA2*, *ATM*, *MLH1, MSH2, APC*, and *TP53*. An important consequence of these groundbreaking discoveries was that many more physicians and patients became aware of the significance of hereditary cancer syndromes. This awareness led to the greater recognition of high-risk families, driving the establishment of clinical genetic centers to address the increased demand for counseling and genetic testing. Concurrently, registries were set up to facilitate early detection and monitor surveillance investigations. While screening of the general population for pancreatic cancer remained unfeasible, in the late 1990s targeted surveillance of high-risk individuals emerged as a potential strategy and the first surveillance initiatives were launched for families predisposed to pancreatic cancer.

## Surveillance for pancreatic cancer: a historical perspective

### Familial pancreatic cancer

In 1973, MacDermott and Kramer described a remarkable family that included four siblings affected by pancreatic cancer, likely representing the first description of familial pancreatic cancer in the medical literature [4]. Following this landmark observation, a series of anecdotal case reports emerged, further highlighting the role of familial factors in the etiology of pancreatic cancer [5–12].

In 1991, Ghadirian et al. reported their case/control study that found having a family history of pancreatic cancer was significantly more common in subjects with pancreatic cancer than in controls [13]. The establishment of the National Familial Pancreas Tumor Registry (NFPTR) in 1994 at Johns Hopkins, Baltimore, through the pioneering efforts of Hruban and others, marked a significant step forward [14, 15], eventually leading to the implementation of pancreatic surveillance programs for at-risk families.

**Fig. 1:**
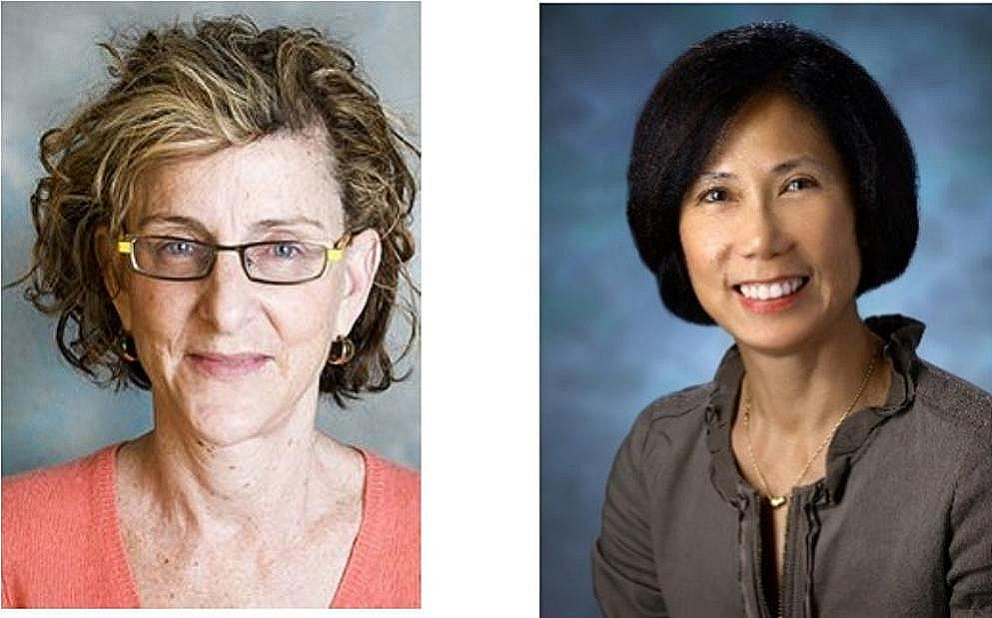
Theresa Brentnall (l) and Marcia Irene Canto (r) whose contributions have been vital to the evolution of familial pancreatic cancer surveillance

Twenty-five years ago, in 1999, Brentnall at the University of Washington, Seatle [16] (Fig. 1) was the first to report the outcomes of surveillance of individuals at risk of pancreatic cancer. During a meeting of the American Pancreatic Association (APA), she presented the compelling case of a 40-year-old asymptomatic Caucasian man whose grandfather, father, four of five paternal uncles, and three first cousins had all died from pancreatic cancer. CT-scanning showed no abnormalities. Opinions on management varied widely among the APA members [17]. Brentnall opted for an extensive surveillance regimen as outlined in her report in this issue encompassing various modalities including EUS, MRI, CT, ERCP, CEA and CA19.9 [18]. The surveillance program identified suspected pancreatic lesions in seven out of 14 high-risk individuals, all of whom underwent total pancreatectomy revealing benign precursor lesions.

In 2004, Canto, at Johns Hopkins, reported another significant milestone: the successful detection of pancreatic cancer through surveillance (Fig. 1) [19]. This study, which involved 37 individuals with familial pancreatic cancer and one patient with Peutz-Jeghers syndrome, demonstrated the effectiveness of EUS in detecting a spectrum of pancreatic lesions ranging from invasive ductal adenocarcinoma to non-neoplastic lesions. Subsequent reports by Canto [20–22] have further underscored the effectiveness of surveillance. The latest update of the original series in 2018 documented 14 pancreatic cancers and 10 high-grade dysplastic lesions in 354 patients. Since 2009, the outcomes of surveillance for familial pancreatic cancer from countries other than the United States have been published, as delineated by Overbeek et al. and Bogdanski et al. in this issue [23, 24]. In 2010, Canto and others established the International Cancer of the Pancreas Screening (CAPS) Consortium, a global initiative aimed at organizing and standardizing pancreatic cancer screening efforts [25]. This consortium has played a pivotal role in advancing the understanding and management of familial pancreatic cancer through collaborative research and clinical initiatives [26].

## Hereditary pancreatic cancer

In the late 1990s and early 2000s, surveillance programs were initiated in families with an underlying pathogenic germline variants. One such program, launched in 2000 at Leiden University Medical Center (LUMC) in the Netherlands, targeted families with Familial Atypical Multiple Mole Melanoma (FAMMM) [27]. Just a few miles from the LUMC is the coastal village of Katwijk, where, three centuries ago, an individual with a genetic predisposition for melanoma settled. As a very stable community, over time the number of carriers of the deleterious gene gradually increased. The responsible gene defect in these families, a pathogenic variant in *CDKN2A* now known as the p16-Leiden founder variant, was later identified by Gruis et al. [28].

**Fig. 2:**
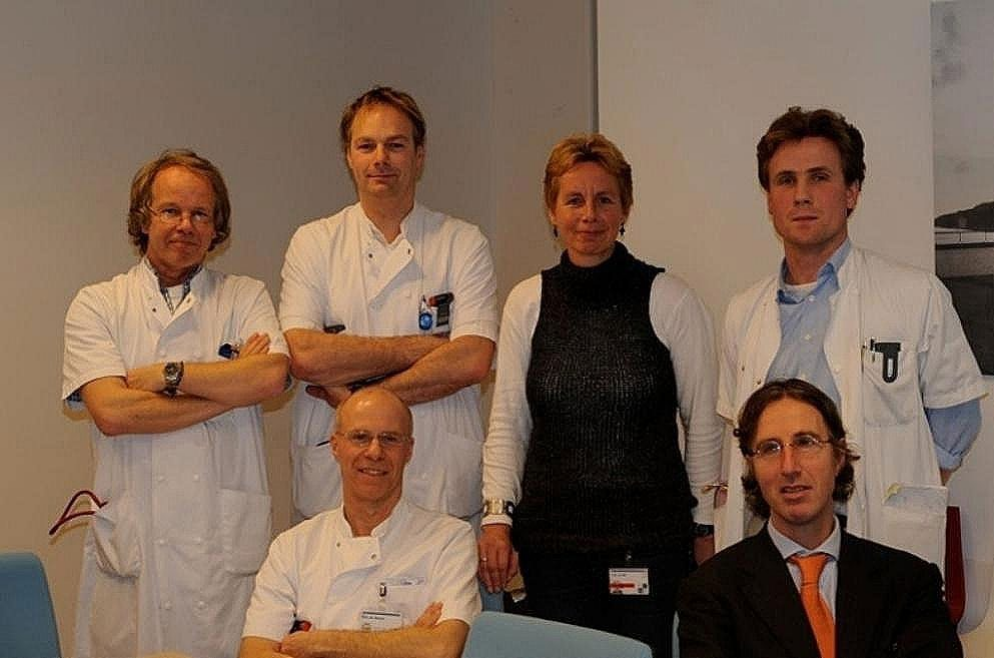
Multidisciplinary Team Surveillance *CDKN2A*-p16-Leiden variant carriers at Leiden University Medical Center, Leiden, The Netherlands (2003); *Upper row*: Hans Vasen (internist), Bert Bonsing (surgeon), Anneke van Mil (genetic counselor), Wouter de Vos tot Nederveen Cappel (gastroenterologist); *Lower row*: Martin Wasser (radiologist), Hans Morreau (pathologist)

In the 1980s, the dermatologist Bergman established a dedicated outpatient department at LUMC to facilitate the early detection of melanoma in individuals with this predisposition [29, 30]. Collaborating with Lynch, Watson and others in 1990, she conducted an evaluation of the tumor spectrum in nine extensive melanoma families [31]. This study revealed a higher than expected incidence of pancreatic cancers - an association further documented in subsequent reports [32, 33]. In 1999, the lifetime risk of pancreatic cancer in carriers of a *CDKN2A* pathogenic variant was calculated to be 17% by age 70 [34]. This significant risk, combined with the groundbreaking paper by Brentnall, prompted Vasen and his team (Fig. 2) to launch a prospective surveillance program for these high-risk individuals in Leiden the following year. A decade later, they reported their experience with surveillance of this cohort; 7 pancreatic cancers detected in 79 carriers in the initial 2011 report [35] and 14 tumors in 178 carriers in a 2016 report [36]. The latest update in 2022 by Klatte reported 36 cancers in 347 *CDKN2A* PGV carriers, currently the largest collection of this carrier group worldwide [37].

## Improved outcomes through surveillance

After 25 years of surveillance of high-risk individuals using established protocols, improved outcomes have finally been demonstrated. Surveillance-detected pancreatic cancers are more likely to be smaller, resectable, and lower stage. Moreover, analyses of long-term overall survival and disease-specific mortality in the *CDKN2A/*p16-Leiden and American CAPS cohorts [38–40] have also recently demonstrated a survival benefit, even after accounting for potential lead-time bias.

## Content and aim of special issue

This Special Issue of Familial Cancer presents a collection of 18 articles authored by leading experts in the field. Under guidance of guest editors Mimi Canto and Michael Goggins, a diverse range of topics related to inherited pancreatic cancer was selected, extending from genetics and pathology to surveillance, surgical intervention, patient perspectives and registries. The primary objective of this issue is to improve the quality of care for high-risk individuals, with a specific focus on refining surveillance methodology to ensure early detection and intervention.

Prior to initiating pancreatic cancer surveillance, it is essential to delineate the high-risk groups eligible for targeted preventive measures. In this issue, Jacobs et al. [3] provide a comprehensive review of hereditary cancer syndromes associated with an increased susceptibility to pancreatic cancer. Additionally, the authors explore potential etiologies of unexplained familial pancreatic cancer and the significance of polygenic risk scores. The authors conclude with a concise overview of environmental and lifestyle factors contributing to pancreatic cancer risk.

Cristina-Marianini-Rios et al. [41] consider a genetic tool to evaluate the clustering of familial pancreatic cancer (FPC) when no pathogenic variant is identified, which is the case in most families. In a large cohort of 125 Spanish FPC families, the authors employed the Best Linear Unbiased Prediction (BLUP) methodology, which estimates an individual’s predicted risk of developing cancer. Their findings could have important implications for general practice.

At present, genetic testing is advised for all patients with pancreatic cancer, as identifying a pathogenic variant has significant implications for genetic counseling, targeted therapies, cascade testing, and appropriate cancer screening approaches for family members found to have a pathogenic variant. Despite the potential clinical value of such gene testing, in most centers only a minority of patients with pancreatic cancer undergo genetic testing. To address this disparity, Rodriguez et al. [42] examined the diverse barriers to genetic testing and discussed alternative healthcare delivery modalities to surmount these hurdles. These modalities include, for example, automated workflows, video-based education, telemedicine genetics care with telephone- or video-consultations and direct-to-consumer testing. Additionally, the authors present insights from several trials assessing the efficacy of these innovative approaches in enhancing the uptake of genetic testing among pancreatic cancer patients.

Over the past two decades, an increasing number of families with a *CDKN2A* pathogenic variant have been identified at the LUMC, Leiden, The Netherlands. Individuals carrying a *CDKN2A* pathogenic variant have an increased risk of developing melanoma (~ 70% lifetime), usually starting in their second decade of life, and pancreatic cancer (~ 20% lifetime) from the age of 40 years onwards. Understanding the pathogenic variant carriers perspectives of genetic testing and family planning is essential to ensuring they receive optimal care. Onnekink et al. [43] examined the motivations of *CDKN2A* pathogenic variant carriers and their relatives to seek genetic testing, their reasons for delaying it, and the preferred timing of testing. The authors also explored whether knowledge of carrier status influenced an individual’s family planning decisions. Additionally, they investigated whether confirmed carriers would consider preimplantation genetic testing, an assisted reproductive technique that enables the selection of embryos lacking a particular genetic predisposition.

Precursor lesions of pancreatic cancer are important as they represent potential targets for cancer prevention. Pflüger, Brosens and Hruban [44] provide comprehensive insights into the characteristics of various precursor lesions associated with pancreatic cancer development. They also present current data on the prevalence and features of these lesions in individuals at risk for familial pancreatic cancer, as well as in carriers of various pathogenic germline variants linked to pancreatic cancer. Additionally, they explore the clinical implications of these lesions, addressing questions such as whether precursor lesion morphology can indicate an underlying pathogenic variant, the significance of identifying a second hit in a precursor lesion in patients with a germline variant, and the screening and treatment implications of finding precursor lesions in such patients.

Currently, recommended methods of surveillance include MRI/MRCP and endoscopic ultrasound (EUS). Overbeek et al. explore the role of EUS within surveillance programs, evaluating its strength, limitations, yield, and impact on patients [23] and compare EUS to MRI/MRCP, discussing the pros and cons of each test. Additionally, they provide a list of published studies that have described prospective surveillance programs that utilize EUS, along with their detection rates for neoplastic progressors. The authors emphasize that there is only limited evidence that pancreatic cancer surveillance results in a true survival benefit.

Boekestijn et al. [45] discuss state-of-the-art MRI techniques and sequences for early pancreatic cancer detection. They present the surveillance protocol used in Leiden since 2000 for *CDKN2A* pathogenic variant carriers, along with results before and after protocol improvement in 2020. Finally, they challenge the prevailing notion that EUS is superior in detecting solid lesions.

To further improve the early detection of pancreatic cancer, there is an urgent need for accurate and inexpensive biomarkers. In this issue, Goggins [46] offers a thorough review of the currently available biomarkers including imaging biomarkers, circulating biomarkers, and those found in cyst fluid, pancreatic juice and urine. The ultimate goal of such biomarkers is to detect cancer early enough to improve survival rates. Goggins stresses the importance of testing biomarkers in the study population where their use is intended, specifically patients with stage I pancreatic cancer. Moreover, given the low disease incidence in high-risk groups, he emphasizes the importance of a high-specificity to minimize the risk of false positives.

Bogdanski et al. [24] present a comprehensive review of pancreatic cancer surveillance in high-risk individuals. They discuss the objectives, targets and methods of surveillance. The authors also provide insights regarding the model used in Leiden to determine if surgery is warranted for a suspected pancreatic lesion or, alternatively, if shortening the surveillance interval (3 or 6 months) is more appropriate. Moreover, they offer a summary of the latest meta-analyses and major studies evaluating surveillance outcomes.

Following the detection of a suspected pancreatic lesion, the question arises regarding the necessity of surgery and whether partial resection or total pancreatectomy is warranted. Maurer et al. [47] discuss the absolute and relative indications for surgery based on current guidelines for managing solid and cystic lesions. In the decision-making, especially concerning relative indications for surgery, they stress the importance of considering the individual’s risk for pancreatic cancer, age, comorbidities, life expectancy, compliance and the risk associated with any surgical procedure. Other topics addressed include indications for prophylactic pancreatectomy, the potential benefits of autologous islet transplantation after total pancreatectomy, and pancreas transplantation. Finally, the authors summarize the overall success rate of current screening programs in achieving surveillance goals in high-risk individuals, specifically, detecting stage I pancreatic cancer or high-risk precursor lesions.

Recent studies demonstrated that surveillance of high-risk individuals leads to higher resection rates and improved survival compared to no surveillance. An additional imperative of an optimal surveillance program is cost-effectiveness. Wang et al. [48] conducted a comprehensive review of available studies that evaluated the cost-effectiveness of various surveillance strategies for familial and hereditary pancreatic cancer in high-risk individuals. The authors identified eight studies with six based on a hypothetical cohort and two based on real data from prospective surveillance cohorts. They summarize detailed results as well as the most influential variables on outcomes such as lifetime risk of pancreatic cancer and surveillance strategies.

Among all the medical reviews featured in this issue, there is a particular touching report from a patient, Amarensia Spruitenburg, a carrier of a pathogenic germline p16-Leiden variant [49]. This report is based on a presentation she delivered a few years ago during a patient meeting. It beautifully captures the array of emotions, ranging from fear to happiness, experienced by patients throughout the process of pancreas surveillance. The positive attitude displayed in her presentation could be a source of inspiration for other patients. Although she successfully overcame her pancreatic tumor, she tragically passed away from an unrelated illness at the end of 2022, nearly six years after undergoing pancreas resection.

As awareness of familial and hereditary cancer grows, new registries are regularly established in various countries. The primary aim of these registries is to promote surveillance in families with a genetic predisposition for cancer. In this issue, the registries for families with familial and hereditary pancreatic cancer established in Japan (2014) [50], Italy (2015) [51] and Spain (2009) [52] are described. The authors outline the criteria for registration, criteria for offering surveillance, surveillance protocols, the coverage of genetic testing and surveillance, registry organization and, if available, the outcome of surveillance.

Matsubayashi et al. present an interesting case of a 73-year-old male [53] who was diagnosed with distal biliary cancer at the age of 56 and more recently with a pancreatic acinar cell carcinoma (PACC) who was found to have a *BRCA2*-pathogenic variant. The patient underwent surgery and received chemotherapy. The authors discuss tumor responses to various treatment regimens, including chemotherapy and olaparib.

As stated in the introduction, screening the general population for pancreatic cancer was not deemed appropriate due to its low prevalence and the lack of accurate surveillance tools. However, with 25 years of experience in the surveillance of high-risk groups, the question inevitably arises: Is this knowledge applicable to the potential screening of the general population? This question will be addressed by Matthias Löhr [54].

In 2023, Gloria Petersen, an pioneering investigator in familial pancreatic cancer passed away. In this issue, Vasen commemorates her significant contributions to the field.

## Conclusion

We extend our sincere gratitude to all authors for their outstanding contributions to this special issue. We believe that this overview of the latest knowledge in the field of inherited pancreatic cancer will assist physicians worldwide in caring for individuals at high-risk. Several reviews in this issue have demonstrated improved survival rates attributed to pancreas surveillance, particularly among families with an underlying pathogenic variant associated with pancreatic cancer. Additionally, over the past decades, the five-year survival rate of patients with sporadic pancreatic cancer has reportedly increased from less than 5% in the 1990s to 12% today (SEER Cancer Statistics: Pancreatic cancer) largely owing to advances in surgery and chemotherapy. The integration of early detection and enhanced management offers hope in the ongoing battle against pancreatic cancer. This is particularly important considering the fact that pancreatic cancer is now the third most common cause of cancer-related deaths in the United States.

## Data Availability

No datasets were generated or analysed during the current study.

## References

[1] Bailar JC, Gornik HL (1997). Cancer undefeated. N Engl J Med.

[2] Flanders TY, Foulkes WD (1996). Pancreatic adenocarcinoma: epidemiology and genetics. J Med Genet.

[3] Jacobs MF, Stoffel EM (2024). Genetic and other risk factors for pancreatic ductal adenocarcinoma (PDAC). Fam Cancer.

[4] MacDermott RP, Kramer P (1973). Adenocarcinoma of the pancreas in four siblings. Gastroenterology.

[5] Friedman JM, Fialkow PJ (1976). Familial carcinoma of the pancreas. Clin Genet.

[6] Dat NM, Sontag SJ (1982). Pancreatic carcinoma in brothers. Ann Intern Med.

[7] Danes BS, Lynch HT (1982). A familial aggregation of pancreatic cancer. An in vitro study. JAMA.

[8] Grajower MM (1983). Familial pancreatic cancer. Ann Intern Med.

[9] Ehrenthal D, Haeger L, Griffin T, Compton C (1987). Familial pancreatic adenocarcinoma in three generations. A case report and a review of the literature. Cancer.

[10] Lynch HT, Fitzsimmons ML, Smyrk TC (1990). Familial pancreatic cancer: clinicopathologic study of 18 nuclear families. Am J Gastroenterol.

[11] Lynch HT, Fusaro L, Lynch JF (1992). Familial pancreatic cancer: a family study. Pancreas.

[12] Reimer RR, Fraumeni JF, Ozols RF, Bender R (1977). Pancreatic cancer in father and son. Lancet.

[13] Ghadirian P, Boyle, Simard A, Baillargeon J, Maisonneuve P, Perret C (1991). Reported family aggregation of pancreatic cancer within a population-based case-control study in the Francophone community in Montreal, Canada. Int J Pancreatol.

[14] Lumadue JA, Griffin CA, Osman M, Hruban RH (1995). Familial pancreatic cancer and the genetics of pancreatic cancer. Surg Clin North Am.

[15] Hruban RH, Petersen GM, Goggins M (1999). Familial pancreatic cancer. Ann Oncol.

[16] Brentnall TA, Bronner MP, Byrd DR, Haggitt RC, Kimmey MB (1999). Early diagnosis and treatment of pancreatic dysplasia in patients with a family history of pancreatic cancer. Ann Intern Med.

[17] Steinberg WM, Barkin J, Bradley EL, DiMagno E, Layer P (1999). Workup of a patient with familial pancreatic cancer. Pancreas.

[18] Brentnall TA (2024). Familial pancreatic cancer: a long fruitful journey. Fam Cancer.

[19] Canto MI, Goggins M, Yeo CJ (2004). Screening for pancreatic neoplasia in high-risk individuals: an EUS-based approach. Clin Gastroenterol Hepatol.

[20] Canto MI, Goggins M, Hruban RH et al (2006) Screening for early pancreatic neoplasia in high-risk individuals: a prospective controlled study. Clin Gastroenterol Hepatol 4(6) 766 – 81; quiz 665. 10.1016/j.cgh.2006.02.005 10.1016/j.cgh.2006.02.005 16682259

[21] Canto MI, Hruban RH, Fishman EK (2012). Frequent detection of pancreatic lesions in asymptomatic high-risk individuals. Gastroenterology.

[22] Canto MI, Almario JA, Schulick RD et al (2018) Risk of Neoplastic Progression in Individuals at High Risk for Pancreatic Cancer Undergoing Long-term Surveillance. Gastroenterology 155(3): 740 – 51 e2 10.1053/j.gastro.2018.05.035 10.1053/j.gastro.2018.05.035 PMC6120797 29803839

[23] Overbeek KA, Cahen DL, Bruno MJ (2024). The role of endoscopic ultrasound in the detection of pancreatic lesions in high-risk individuals. Fam Cancer.

[24] Bogdanski AM, van Hooft JE, Boekestijn B (2024). Aspects and outcomes of surveillance for individuals at high-risk of pancreatic cancer. Fam Cancer.

[25] Canto MI, Harinck F, Hruban RH (2013). International Cancer of the pancreas Screening (CAPS) Consortium summit on the management of patients with increased risk for familial pancreatic cancer. Gut.

[26] Goggins M, Overbeek KA, Brand R (2020). Management of patients with increased risk for familial pancreatic cancer: updated recommendations from the International Cancer of the pancreas Screening (CAPS) Consortium. Gut.

[27] de Vos tot Nederveen, Cappel WH, Lagendijk MA, Lamers CB, Morreau H, Vasen HF (2003) Surveillance for familial pancreatic cancer. Scand J Gastroenterol Suppl 23994–99. 10.1080/00855920310002762 10.1080/00855920310002762 14743890

[28] Gruis NA, van der Velden PA, Sandkuijl LA (1995). Homozygotes for CDKN2 (p16) germline mutation in Dutch familial melanoma kindreds. Nat Genet.

[29] Bergman W, Palan A, Went LN (1986). Clinical and genetic studies in six Dutch kindreds with the dysplastic naevus syndrome. Ann Hum Genet.

[30] Vasen HF, Bergman W, van Haeringen A, Scheffer E, van Slooten EA (1989). The familial dysplastic nevus syndrome. Natural history and the impact of screening on prognosis. A study of nine families in the Netherlands. Eur J Cancer Clin Oncol.

[31] Bergman W, Watson P, de Jong J, Lynch HT, Fusaro RM (1990). Systemic cancer and the FAMMM syndrome. Br J Cancer.

[32] Goldstein AM, Fraser MC, Struewing JP (1995). Increased risk of pancreatic cancer in melanoma-prone kindreds with p16INK4 mutations. N Engl J Med.

[33] Whelan AJ, Bartsch D, Goodfellow PJ (1995). Brief report: a familial syndrome of pancreatic cancer and melanoma with a mutation in the CDKN2 tumor-suppressor gene. N Engl J Med.

[34] Vasen HF, Gruis NA, Frants RR, van Der Velden PA, Hille ET, Bergman W (2000). Risk of developing pancreatic cancer in families with familial atypical multiple mole melanoma associated with a specific 19 deletion of p16 (p16-Leiden). Int J Cancer.

[35] Vasen HF, Wasser M, van Mil A (2011). Magnetic resonance imaging surveillance detects early-stage pancreatic cancer in carriers of a p16-Leiden mutation. Gastroenterology.

[36] Vasen H, Ibrahim I, Ponce CG (2016). Benefit of Surveillance for Pancreatic Cancer in High-Risk individuals: outcome of long-term prospective Follow-Up studies from three European Expert centers. J Clin Oncol.

[37] Klatte DCF, Boekestijn B, Wasser M (2022). Pancreatic Cancer surveillance in carriers of a germline CDKN2A pathogenic variant: yield and outcomes of a 20-Year prospective Follow-Up. J Clin Oncol.

[38] Klatte DCF, Boekestijn B, Onnekink AM et al (2023) Surveillance for Pancreatic Cancer in High-Risk Individuals Leads to Improved Outcomes: A Propensity Score-Matched Analysis. Gastroenterology 164(7): 1223-31 e4 10.1053/j.gastro.2023.02.032 10.1053/j.gastro.2023.02.032 36889551

[39] Dbouk M, Katona BW, Brand RE (2022). The Multicenter Cancer of pancreas Screening Study: Impact on Stage and Survival. J Clin Oncol.

[40] Blackford AL, Canto MI, Dbouk M et al (2024) Surveillance of High-Risk Individuals Improves Pancreatic Cancer Survival. JAMA Oncology, in press 10.1001/jamaoncol.2024.1930 PMC11223057 38959011

[41] Cristina-Marianini-Rios, Castillo Sanchez M, Garcia Garcia de Paredes Aea (2024) The Best Linear Unbiased Prediction (BLUP) method as a tool to estimate the lifetime risk of pancreatic ductal adnocarcinoma in high-risk individuals with no known pathogenic germline variants. Familial Cancer. 10.1007/s10689-024-00397-w 10.1007/s10689-024-00397-w PMC11254992 38780705

[42] Rodriguez NJ, Syngal S (2024). Expanding access to genetic testing for pancreatic cancer. Fam Cancer.

[43] Onnekink AM, Klatte DCF, van Hooft JE et al (2024) Attitudes toward genetic testing, family planning and preimplantation genetic testingin families with a germline *CDKN2A* pathogenic variant. Familial Cancer. in press 10.1007/s10689-024-00401-3 PMC11255069 38822936

[44] Pfluger MJ, Brosens LAA, Hruban RH (2024). Precursor lesions in familial and hereditary pancreatic cancer. Fam Cancer.

[45] Boekestijn B, Feshtali S, Vasen H (2024). Screening for pancreatic cancer in high-risk individuals using MRI: optimization of scan techniques to detect small lesions. Fam Cancer.

[46] Goggins M (2024). The role of biomarkers in the early detection of pancreatic cancer. Fam Cancer.

[47] Maurer E, Bartsch DK (2024). Surgical aspects related to hereditary pancreatic cancer. Fam Cancer.

[48] Wang L, Levinson R, Mezzacappa C, Katona BW (2024) A review of the cost-effectiveness on surveillance for hereditary pancreatic cancer. Familial Cancer. 10.1007/s10689-024-00392-1 10.1007/s10689-024-00392-1 PMC11255025 38795221

[49] Spruitenburg AM, Vasen HF (2024). The odyssee from surveillance to the detection of pancreatic cancer, total pancreatectomy, and its impact on life. Insights from a p16-Leiden pathogenic variant carrier. Fam Cancer.

[50] Matsubayashi H, Morizane C (2024). Familial and hereditary pancreatic cancer in Japan. Fam Cancer.

[51] Archibugi L, Casciani F, Carrara S (2024). The Italian registry of families at risk for pancreatic cancer (IRFARPC): implementation and evolution of a national program for pancreatic cancer surveillance in high-risk individuals. Fam Cancer.

[52] Earl J, Fuentes R, Sanchez MEC (2024). The Spanish Familial Pancreatic Cancer Registry (PANGENFAM): a decade follow-up of individuals at high-risk for pancreatic cancer. Fam Cancer.

[53] Matsubayashi H, Todaka A, Tsushima T (2024). The response of pancreatic acinar cell carcinoma to platinum and olaparib therapy in a germline BRCA2 variant carrier: case report and literature review. Fam Cancer.

[54] Lohr JM, Ohlund D, Soreskog E et al (2024) Can our experience with surveillance for inherited pancreatic cancer help to identify early pancreatic cancer in the general population? Fam Cancer. 10.1007/s10689-024-00363-6 10.1007/s10689-024-00363-6 PMC11255073 38441833

